# Sealing ability of AH Plus endodontic sealer, seal apex, multi-walled carbon nanotube-incorporated AH plus and carbon nanotube-incorporated seal apex using dye penetration microleakage

**DOI:** 10.6026/9732063002001154

**Published:** 2024-09-30

**Authors:** Hrishikesh A Saoji, Dinesh Rao, Sandeep Metgud

**Affiliations:** 1 Pacific University of Health Sciences, Udaipur, Rajasthan, India; 2 Department of Pediatric Dentistry, Pacific Dental College, Udaipur, Rajasthan, India; 3 Department of Conservative Dentistry and Endodontics, Pacific Dental College, Udaipur, Rajasthan, India

**Keywords:** Multi-walled carbon nanotubes, root canal sealers, microleakage, endodontic treatment

## Abstract

This study evaluates the sealing ability of multi-walled carbon nanotube (MWCNT)-incorporated root canal sealers in the apical third of the root canal. Fifty extracted single-rooted human teeth were divided into five groups: control (no sealer), MWCNT-incorporated AH Plus, MWCNT-incorporated Seal Apex, AH Plus and Seal Apex. The sealing ability was assessed through dye penetration microleakage. Results indicated that the MWCNT-incorporated sealers exhibited significantly less microleakage compared to traditional sealers, with MWCNT-incorporated AH Plus showing the least leakage. The control group exhibited the highest microleakage. MWCNTs enhanced the sealing efficacy by improving sealer adaptation to the canal walls. These findings highlight the potential of carbon nanotube-enhanced sealers to improve endodontic treatment outcomes. Further studies incorporating in vivo models and longer evaluation periods are recommended to confirm the clinical applicability of these advanced materials.

## Background:

Root canal treatment is crucial for eliminating microbial presence within the canal system and restoring the root structure three-dimensionally to ensure the long-term retention of teeth. The effectiveness of this treatment hinges significantly on the adequacy of the root canal filling material, as inadequate filling can induce inflammatory responses leading to treatment failure. An essential component in this context is the endodontic sealer, which is instrumental in filling microgaps, sealing accessory canals and bonding the core material to the root canal walls, thus entrapping any remaining microorganisms [1]. The sealing ability of the endodontic sealer is paramount, as it directly influences microleakage, which is a critical determinant of the success of root canal treatments. Over the years, the formulation of root canal sealers has evolved, transitioning from traditional zinc oxide eugenol-based sealers to contemporary methacrylate resin-based sealers, such as Super-Bond RC [2]. Among the various sealers, AH Plus has garnered attention for its low solubility, excellent dimensional stability and adhesive properties to root dentin due to its creep behaviour [3, 4]. The material has shown promising results in terms of microleakage and sealing ability, although it is associated with a major drawback of a long setting time [5]. AH-26, a variant of AH Plus, has demonstrated significantly lower dye penetration compared to other sealers [6]. A recent systematic review has identified controversial evidence in favour and against the material [7]. These findings underscore the on-going need for advancements in sealer formulations to enhance their sealing efficacy. Recent advancements in materials science have focused on improving the antibacterial properties, physical characteristics, mechanical performance and sealing effectiveness of resin-based root canal sealers. Studies have explored the incorporation of nanoparticles to enhance these properties [3]. For instance, Carvalho *et al.* (2021) examined the incorporation of chlorhexidine-hexametaphosphate nanoparticles into root canal sealers, while Afkhami *et al.* (2021) evaluated the antibacterial effectiveness of silver nanoparticle-modified AH Plus sealer [8, 9]. Moreover, Ratih *et al.* (2020) concluded that integrating chitosan nanoparticles into epoxy resin sealers significantly enhances their apical sealing capacity [10].

Nanoparticles possess the ability to penetrate dentinal tubules, thereby offering a promising approach to improving the sealing ability of new-generation endodontic sealers. Among these, carbon nanotubes and graphene stand out due to their unique electrical, mechanical, and physical properties [11-14]. Carbon nanotubes, essentially cylindrical sheets of graphene and graphene-based materials have demonstrated various dental applications. These include usage in fabricating scaffolds for tissue engineering, promoting osteogenic differentiation, periodontal tissue regeneration, dental pulp regeneration, and as antimicrobial fillers in polymer dental adhesives and cements [11, 12]. The potential of graphene oxide-based endodontic sealers was highlighted by Nizami *et al.* (2022), who conducted an in vitro study demonstrating that these sealers offered excellent sealing and antimicrobial properties, indicating their potential to enhance the success rates of endodontic treatments and regenerative procedures [14]. Despite the significant progress in developing advanced root canal sealers, there remains a gap in the literature regarding the comparative effectiveness of carbon nanotube-incorporated sealers versus traditional and other nanoparticle-enhanced sealers. This study addresses this gap by evaluating and comparing the sealing ability of carbon nanotube-incorporated root canal sealers, focusing specifically on their performance in the apical third of the root canal. Specific objectives include evaluating the sealing ability of AH Plus endodontic sealer, Seal Apex, multi-walled carbon nanotube-incorporated AH Plus and carbon nanotube-incorporated Seal Apex using dye penetration microleakage, alongside a control group with non-sealer used teeth.

## Materials and Methods:

## Sample selection and preparation:

Fifty extracted human single-rooted teeth with a single canal and Vertucci class I canal anatomy were selected for the study. Root canal treatment was initiated with access opening, followed by canal negotiation using a 10-15 size K instrument. The working length was determined radiographically. Cleaning and shaping were performed using rotary ProTaper Gold instruments (Sx, S1, S2, F1, F2, and F3) and an X-Smart endomotor set at 300 RPM with a torque of 2.6. Copious irrigation with 5.25% NaOCl and saline was conducted. Master cone selection involved using F3 size ProTaper gutta-percha cones, positioned 0.5 mm short of the radiographic apex. The canals were dried using F3 size paper points.

## Experimental groups:

The teeth were randomly divided into five groups to receive obturation by cold lateral technique with F3 gutta-percha cones and the predetermined sealers:

Group A: No sealer (control)

Group B: Multi-walled carbon nanotube-incorporated AH Plus sealer

Group C: Multi-walled carbon nanotube-incorporated Seal Apex sealer

Group D: AH Plus sealer

Group E: Seal Apex sealer

## Preparation of sealers:

Multi-walled carbon nanotubes (MWCNTs) were commercially purchased from Ultra Nanotech Pvt. Ltd, India. These were prepared using the chemical vapor deposition method, with a length of 2-20 µm, a diameter of 10-15 nm, and a purity of 97%. AH Plus and Seal Apex were the root canal sealers used in this study. The preparation of carbon nanotube-incorporated sealers involved adding 1 wt% of MWCNT powder to AH Plus and Seal Apex sealers. The MWCNT powder was mixed with the sealers using a mechanical amalgamator at 300 RPM for 30 seconds. The samples were stored in a microbiological incubator at 37°C with 100% humidity for seven days. Nail varnish was applied to the root surface of the samples, excluding the apical 3 mm. The samples were then immersed in 2% methylene blue dye for 24 hours. Subsequently, the samples were sectioned longitudinally using a diamond disc. Analysis was conducted using a microscope under 30X magnification.

## Statistical analysis:

Inter-group comparisons were performed using a one-way ANOVA test. Pair-wise comparisons were done by the Post hoc Tukey test. A value of p<0.05 was considered as statistically significant.

## Results:

**Figure 1:**
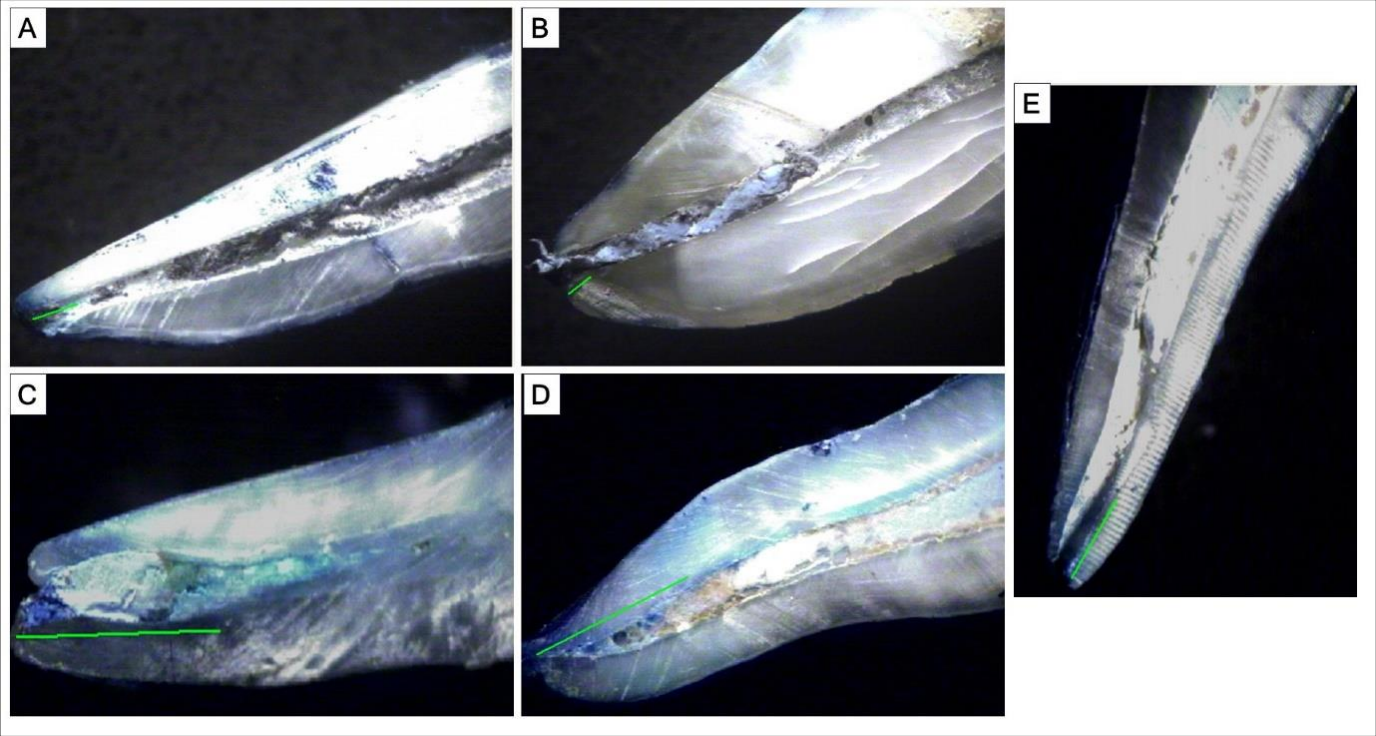
Stereoscopic image (4.5x magnification) of A) MWCNTS+ Seal apex; B) MWCNTS+ AH Plus; C) AH Plus; D) Seal apex; E) No Sealer

The results showed that the least extent of microleakage was seen in MWCNTS AH plus, followed by MWCNTS Seal Apex (Figure 1). The maximum extent of microleakage was seen in the control group (2.73 mm) with no sealer. Overall, the difference in microleakage among the five groups was statistically significant.

Pairwise Comparison of microleakage showed that there was no significant difference (p>0.05) in microleakage between MWCNTS AH plus and MWCNTS seal apex (Table 1). The difference between MWCNTS AH plus sealer and AH plus seal apex and control group was statistically significant (p<0.05) also difference between MWCNTS seal apex, AH plus seal apex & control group was statically significant. AH plus sealer showed significantly less (p<0.05) microleakage as compared to seal apex and control group. Seal apex showed significantly less (p<0.05) microleakage when compared with control group.

## Discussion:

The findings of this study underscore the significant impact of incorporating MWCNTs into traditional endodontic sealers on their sealing efficacy, specifically in the apical third of root canals. The reduction in microleakage observed with MWCNT-incorporated AH Plus and Seal Apex sealers highlights the potential of these advanced materials to enhance the success rates of root canal treatments. The unique properties of MWCNTs, such as their high surface area, mechanical strength and ability to penetrate dentinal tubules, likely contribute to this enhanced performance. These properties facilitate better adaptation and bonding of the sealer to the canal walls, thereby reducing the potential for microleakage. Previous studies have demonstrated similar benefits with other nanoparticle-incorporated materials, emphasizing the role of nanoparticles in improving the physical and mechanical properties of dental sealers [15].

The superior sealing ability of the MWCNT-incorporated sealers compared to their traditional counterparts, AH Plus and Seal Apex, without MWCNTs, further emphasizes the advantages of nanoparticle incorporation. The control group, which did not use any sealer, exhibited the highest extent of microleakage, reaffirming the critical role of sealers in preventing leakage and subsequent treatment failure. Among the traditional sealers, AH Plus showed significantly less microleakage than Seal Apex, consistent with previous studies highlighting AH Plus's superior sealing properties due to its excellent dimensional stability and adhesive characteristics [16]. This aligns with earlier research highlighting the importance of dimensional stability and low solubility in achieving effective sealing in root canal treatments [17]. Interestingly, no significant difference in microleakage was observed between the MWCNT-incorporated AH Plus and MWCNT-incorporated Seal Apex groups. This suggests that the incorporation of MWCNTs effectively enhances the sealing ability of both types of sealers to a comparable extent. The significant reduction in microleakage with MWCNT-incorporated sealers compared to traditional sealers indicates that these advanced materials could offer a reliable solution for improving the outcomes of root canal treatments. Previous studies on nanoparticle-modified sealers have shown similar improvements in sealing ability and antimicrobial properties [18].

The superior sealing ability of the MWCNT-incorporated sealers compared to their traditional counterparts, AH Plus and Seal Apex, without MWCNTs, further emphasizes the advantages of nanoparticle incorporation. The control group, which did not use any sealer, exhibited the highest extent of microleakage,reaffirming the critical role of sealers in preventing leakage and subsequent treatment failure. Among the traditional sealers, AH Plus showed significantly less microleakage than Seal Apex, consistent with previous studies highlighting AH Plus's superior sealing properties due to its excellent dimensional stability and adhesive characteristics [16]. This aligns with earlier research highlighting the importance of dimensional stability and low solubility in achieving effective sealing in root canal treatments [17]. Interestingly, no significant difference in microleakage was observed between the MWCNT-incorporated AH Plus and MWCNT-incorporated Seal Apex groups. This suggests that the incorporation of MWCNTs effectively enhances the sealing ability of both types of sealers to a comparable extent. The significant reduction in microleakage with MWCNT-incorporated sealers compared to traditional sealers indicates that these advanced materials could offer a reliable solution for improving the outcomes of root canal treatments. Previous studies on nanoparticle-modified sealers have shown similar improvements in sealing ability and antimicrobial properties [18].

Earlier studies have also demonstrated the benefits of incorporating nanoparticles into endodontic sealers to improve their physical and antimicrobial properties. For instance, studies on silver nanoparticle-modified sealers and chitosan nanoparticle-incorporated epoxy resin sealers have shown promising results in enhancing sealing ability and antimicrobial efficacy [19,20]. The current study adds to this body of knowledge by specifically evaluating the impact of MWCNTs, known for their unique mechanical and physical properties, on the sealing performance of root canal sealers. The potential of MWCNTs in dental applications extends beyond endodontic sealers, as they have been explored in various dental materials and applications, including scaffolds for tissue engineering and antimicrobial dental adhesives [20, 21]. Despite the promising results, this study has several limitations that need to be addressed. Firstly, the in vitro nature of the study does not fully replicate the complex clinical conditions encountered in vivo, such as the presence of blood, saliva and varying pH levels, which can affect the sealing ability and longevity of the endodontic sealers. Additionally, the use of dye penetration as a method to evaluate microleakage, while widely accepted has its limitations in terms of sensitivity and reproducibility. Another limitation is the relatively short incubation period of seven days, which may not adequately reflect the long-term performance and stability of the sealers. Moreover, the study did not evaluate the potential cytotoxicity or biocompatibility of the MWCNT-incorporated sealers, which are crucial factors for their clinical application. Future studies should incorporate longer observation periods, in vivo models and comprehensive biocompatibility assessments to better understand the clinical implications of using MWCNT-enhanced sealers. Overall, the present study contributes to a growing body of evidence that supports the use of advanced nanotechnology in endodontics to improve treatment efficacy and patient outcomes.

## Figures and Tables

**Table 1:** Intergroup and pairwise comparisons of microleakage

**Groups**	**Mean **	**SD**	**F value**	**P value **	**Pairwise comparison**
MCNTS + sealapex (GSP)	0.69	0.26			GSP = GAH; GSP< SP, AH, CGAH < SP, AH, CAH < SP, CSP < C
MCNTS + AH plus (GAH)	0.57	0.15			
Seal apex (SP)	2.02	0.41	63.469	<0.001*	
AH plus (AH)	1.61	0.58			
Control (C)	2.73	0.24			
